# Effects of basic carbohydrate counting versus standard dietary care for glycaemic control in type 2 diabetes (The BCC Study): a randomised, controlled trial

**DOI:** 10.1038/s41387-024-00307-0

**Published:** 2024-06-27

**Authors:** Bettina Ewers, Martin B. Blond, Jens M. Bruun, Tina Vilsbøll

**Affiliations:** 1 Department of Clinical and Translational Research, Steno Diabetes Center Copenhagen, Herlev, Denmark; 2 Steno Diabetes Center Aarhus, Aarhus, Denmark; 3 Department of Clinical Medicine, University of Aarhus, Aarhus, Denmark; 4 Faculty of Health and Medical Sciences, University of Copenhagen, Copenhagen, Denmark

**Keywords:** Type 2 diabetes, Patient education, Nutrition

## Abstract

**Background:**

Clinical guidelines recommend basic carbohydrate counting (BCC), or similar methods to improve carbohydrate estimation skills and to strive for higher consistency in carbohydrate intake potentially improving glycaemic control. However, evidence for this approach in type 2 diabetes (T2D) is limited.

**Objective:**

To examine the efficacy of a structured education program in BCC as add-on to standard dietary care on glycaemic control in individuals with T2D.

**Methods:**

The BCC Study was a randomized, controlled, open-label, parallel-group trial. Individuals with T2D aged 18-75 years with glycated haemoglobin A1c (HbA1c) 53–97 mmol/mol (7.0–11.0%) were randomly assigned (1:1) to BCC or standard dietary care. The primary outcomes were differences in changes in HbA1c or glycaemic variability (calculated as mean amplitude of glycaemic excursions [MAGE]) between groups after six months of intervention.

**Results:**

Between September 2018 and July 2021, 48 participants were randomly assigned, 23 to BCC and 25 to standard dietary care. Seven participants did not receive the allocated intervention. From a baseline-adjusted mean of 65 mmol/mol (95% CI 62-68 [8.1%, 7.8-8.4]), HbA1c changed by −5 mmol/mol (−8 to −1 [−0.5%, −0.7 to −0.1]) in BCC and -3 mmol/mol (−7 to 1 [−0.3%, −0.6 to 0.1]) in standard care with an estimated treatment effect of −2 mmol/mol (−7 to 4 [−0.2%, −0.6 to 0.4]); *p* = 0.554. From a baseline-adjusted mean of 4.2 mmol/l (3.7 to 4.8), MAGE changed by −16% (−33 to 5) in BCC and by −3% (−21 to 20) in standard care with an estimated treatment effect of −14% (−36 to 16); *p* = 0.319. Only median carbohydrate estimation error in favour of BCC (estimated treatment difference −55% (−70 to −32); *p* < 0.001) remained significant after multiple testing adjustment.

**Conclusions:**

No glycaemic effects were found but incorporating BCC as a supplementary component to standard dietary care led to improved skills in estimating carbohydrate intake among individuals with T2D.

## Introduction

Type 2 diabetes (T2D) is a chronic condition requiring continuous self-management, beyond regular healthcare visits. Consequently, evidence-based education with specific focus on diet and nutrition is crucial for empowering individuals to effectively manage their diabetes [1]. Giving priority to dietary management is essential for preventing the onset of diabetes-related complications and improving quality of life [1, 2]. A variety of eating patterns have been recommended by the European and American diabetes associations [2, 3]. Additionally, a recent network meta-analysis found that several dietary approaches (including low-fat, vegetarian, Mediterranean, high-protein, moderate-carbohydrate, low-carbohydrate, low glycaemic index/loads, paleolithic diets) improve glycaemic control in T2D [4]. Compared with various eating patterns, carbohydrate counting is an approach to manage carbohydrate amounts and balance carbohydrate intake at meals (i.e., basic carbohydrate counting [BCC]). Advanced carbohydrate counting has been recommended for several decades, primarily benefiting individuals with type 1 diabetes (T1D), but also showing positive effects in T2D in combination with basal-bolus insulin regimes [2, 5, 6]. Despite the impact of the total carbohydrate intake in a meal on the postprandial glucose response, there remains a scarcity of evidence regarding the effectiveness of BCC as a dietary approach for enhancing glycaemic control [7, 8]. Furthermore, accuracy in carbohydrate estimation is presumed an intermediate marker likely to have an impact on glycaemic control in individuals with diabetes. This has been found in some studies (mainly including individuals with T1D) [9–11] but not others [12–15]. There are multiple educational approaches to BCC including gram counting, exchanges, and experience-based estimation [16–18]. None of which have been thoroughly described or examined in clinical trials. Thus, providing knowledge and practical skills in estimating carbohydrate portions more accurately and self-monitoring carbohydrate intake appears highly relevant. By improving carbohydrate estimation skills and encourage monitoring of carbohydrate intake, aiming at a high day-to-day and meal-to-meal consistency, individuals with T2D may improve their carbohydrate food choices and portion sizes. Ideally, this could result in clinical improvements such as a reduction in plasma glucose variability and time spent with hyperglycaemia thereby improving glycated haemoglobin A1c (HbA1c) as seen in individuals with T1D [19]. The aim of the current study was to examine the efficacy of a structured education program in BCC as add-on to standard dietary care on glycaemic control in individuals with T2D. Our hypothesis was that BCC is superior for improving glycaemia, assessed by HbA1c and mean amplitude of glycaemic excursions (MAGE), when compared with standard dietary care.

## Subjects and methods

### Study design and participants

A protocol paper containing a detailed description of the trial has been published previously [20]. The study was a single-center, parallel-group, randomised, controlled, open-label, superiority trial conducted at the outpatient clinic at Steno Diabetes Center Copenhagen, a tertiary health care facility in the Capital Region of Denmark. The total study duration was 12 months. Inclusion criteria were age 18–75 years, T2D, diabetes duration ≥ 12 months, treated in the outpatient clinic at Steno Diabetes Center Copenhagen, HbA1c 53-97 mmol/mol (7.0–11.0%), treatment with diet or any glycose-lowering medication. Exclusion criteria were ongoing practice of carbohydrate counting, participation in a BCC program within the last two years, use of an automated bolus calculator, low daily carbohydrate intake (defined as below 25 E% or <100 g/day), gastroparesis, uncontrolled medical issues affecting dietary intake/judged unsuitable for participation, pregnancy, plans of pregnancy, breastfeeding, participation in other clinical trials or the inability to understand the information and to give informed consent. The screening and study visits (at baseline, after 6 months intervention and after 6 months follow-up) were carried out by the study personnel. The inclusion period was 28 September 2018 to 12 July 2021. The trial was carried out in accordance with the Helsinki Declaration after approval by the Regional Scientific Ethics Committee of the Capital Region of Denmark (H-18014918). The data collection was performed in accordance with the General Data Protection Regulation (VD-2018-233, I-Suite no.: 6474). The trial is registered at ClinicalTrials.gov (registration no. NCT03623139).

### Screening and randomisation

Individuals with interest in participation attended a screening visit, and those eligible for inclusion were randomised 1:1 to either BCC or control (standard dietary care). Informed consent was obtained from all individuals prior to screening. The randomisation list was generated by an external statistician and uploaded to the electronic data management system REDCap (8.10.18, Vanderbilt University, Tennessee, USA). Participants eligible for inclusion in the study according to the screening were randomised at the end of the screening visit by the study investigator/study personnel using the randomisation module [20].

### Interventions

Participants allocated to standard dietary care received three individual dietary counselling sessions (total duration 2 h; week 0, 2 and 12). All participants in the BCC group also attended a group course (4-8 participants; total duration 8 h; week 2, 4 and 12). The BCC program constituted a structured group-based educational initiative designed to help individuals with T2D manage their blood glucose levels by regulating their carbohydrate intake. Program activities encompassed concise theoretical presentations on food and nutrition in relation to diabetes (participants were not given a complete diabetes self-management program), problem-solving exercises incorporating hands-on group activities to identify carbohydrate sources, and practical sessions involving the measurement and calculation of carbohydrate content in diverse foods. Various approaches to carbohydrate monitoring were explored, including gram counting using nutrition labels, interpretation of carbohydrate tables, utilization of smartphone applications for estimating carbohydrate portion sizes, and improving skills in estimating carbohydrate portion sizes through visual training. Furthermore, participants were instructed to maintain a dietary log during the course, documenting their food intake and blood glucose levels over a 4-day period. The dietary data recorded in the log was used to ascertain total carbohydrate intake and daily variations across meals and snacks and to develop a personal carbohydrate plan with recommendations for daily carbohydrate intake. Finally, the BCC program also encompassed discussions on dietary coping strategies with the integration of peer modeling and support. All group courses and individual dietary counselling sessions were carried out by the same study dietitians who followed the planned curriculum. More details concerning the content in the BCC program and standard dietary care have been reported in the protocol paper [20]. Participants were advised not to change their physical activity pattern during the study period. Changes in medication were allowed during the study period.

### Outcome measures

The primary outcomes were changes in HbA1c and MAGE – a measure of glycaemic variability – from baseline to end-of-treatment at six months. A full list of secondary and exploratory outcomes is described in detail in the protocol paper [20].

### Sample size

The trial was designed to have 80% statistical power (α level of 0.05) to detect a minimal important difference in HbA1c of 3 mmol/mol (0.3%) (SD 7 mmol/mol (0.6%)) between the two groups. This was primarily based on findings from clinical trials of various dietary approaches for reducing HbA1c [4, 21] and considered clinically relevant as part of a multidisciplinary approach for the management of hyperglycaemia in T2D. The clinical target for MAGE in T2D is unknown [22] but the trial was designed to be able to detect a difference in MAGE of ≥ 0.3 mmol/l (SD 0.7 mmol/l) between the groups [20]. Based on these assumptions and accounting for a dropout rate of 30% and subgroup analyses, the sample size was planned to include a total of 226 participants in the trial (113 in each group).

### Changes due to the COVID-19 pandemic

From March 2020 all elective consultations in Denmark were shifted to digital consultations for non-acute patients in response to COVID-19. Since the participants in our trial were primarily overweight, older, and chronically ill with several co-morbidities, they were considered at a high risk of morbidity and mortality if infected. Consequently, enrollment of new participants was discontinued until September 2020 and again from November 2020 to February 2021 due to a new rise in the number of COVID-19 cases in Denmark. Similarly, all planned study visits for enrolled participants prior to COVID-19 were temporarily postponed in the two lock-down periods. Besides the first visit when treatment was initiated, most visits took place later than scheduled during the pandemic with lockdown and particularly the last visit (12-month follow-ups) were scattered over a longer period. Thus, the trial was terminated in July 2021 before reaching the intended sample size without reviewing data beforehand [23].

### Statistical analyses

At the end of the trial, a statistician consultant reviewed our trial protocol and protocol paper [20]. An adjusted statistical analyses plan (SAP) was written, signed by all co-authors, and uploaded at clinicaltrials.gov before data handling and statistical analyses were initiated (see Appendix A). Baseline data are reported as means with SD for normally distributed continuous variables, and medians with 25th and 75th percentiles for non-normally distributed variables and with numbers and percentages for categorical variables. We performed intention-to-treat analyses including all available data to compare the treatment effects between the two groups for the pre-specified primary outcomes HbA1c and MAGE and selected secondary/exploratory outcomes. Treatment effects are presented as the baseline corrected differences between the groups for all outcomes. Outcomes were modeled by linear mixed-effects models. Baseline correction was performed by placing all participants in the control group at baseline. Visit and the interaction between treatment group and visit were included as fixed effects. Assumptions of normality and homogeneity of variances for residuals were assessed with graphical methods before estimating the treatment effects and if necessary, outcomes were log-transformed in the analyses and back-transformed for presentation. Results are presented as estimated mean differences in change (95% CI) between and within groups with two-sided p values. Non-parametric tests (Wilcoxon) were used to compare changes in the summed scores from baseline to end-of-treatment for the three psychometric tests due to non-normal distributions. Statistical significance was inferred at a two-tailed *p* < 0.05. The false discovery rate (FDR) of the secondary/exploratory outcomes were controlled using the method of Benjamini and Hochberg ( < 5% was used as the threshold) [24]. Missing data were handled implicitly by maximum likelihood estimation in the linear mixed model and missing data were assumed to be missing at random. Statistical analyses were conducted in SAS Enterprise Guide software version 8.3 Update 3 (SAS Institute Inc., Cary, North Carolina, USA) and R software version 4.0.2 (R Core Team, R Foundation for Statistical Computing, Vienna, Austria).

## Results

We assessed 252 patients for eligibility: 78 declined our invitation or did not answer, 126 did not meet our inclusion criteria. In total, 48 participants were included and randomly assigned with 23 assigned to the BCC group and 25 assigned to the standard dietary care group (control). The number of participants who dropped out or were lost to follow-up for each group during each phase of the trial has been reported in the flow diagram as supplementary material (Fig. S1). Baseline characteristics of the participants by allocation are shown in Table 1 and supplementary in Table S1. Overall, the study population was around 62 years of age (68% males), with an average diabetes duration of 16 years and moderately uncontrolled glycaemic regulation, HbA1c: 63 mmol/mol (7.9%). A high percentage of participants used various types of antidiabetic medication (especially metformin, glucagon-like peptide-1 receptor agonizts (GLP-1RAs), sodium-glucose co-transporter 2 inhibitors (SGLT-2s), and insulin), antihypertensive and lipid-lowering medication (Table 1). During the intervention period, modifications in prescribed medication and titrations were observed (Table S2–S4). Five participants discontinued use of GLP-1RAs and 4 participants discontinuing use of SGLT-2s in the standard group (Table S2). The average number of visits at the outpatient diabetes clinic during the intervention period were endocrinologist: 1.7 (in BCC) and 1.5 (in standard); nurse: 0.8 (in BCC) and 1.1 (in standard); dietitian: 6.1 (in BCC - including group visits) and 3.2 (in standard). The total average number of visits during the study period including the follow-up period were endocrinologist: 2.5 (in BCC) and 2.3 (in standard); nurse: 1.5 (in BCC) and 1.3 (in standard); dietitian: 6.1 (in BCC) and 3.2 (in standard). Participants did not have any visits with a physiotherapist or exercise physiologist during the study period according to protocol.

**Table 1:** Baseline characteristics.

Characteristics	Overall (*n* = 47^a^)	BCC (*n* = 22^a^)	STANDARD (*n* = 25)
Age (years)	62 (52, 68)	62 (57, 66)	61 (51, 69)
Men, *n* (%)	32 (68%)	15 (68%)	17 (68%)
Self-reported white origin, *n* (%)	45 (96%)	22 (100%)	23 (92%)
Current smoker, *n* (%)	1 (2%)	0 (0%)	1 (0%)
Number of smoking years	25 (20, 37)	33 (15, 42)	25 (20, 32)
Education, *n* (%):			
Elementary school	3 (6%)	1 (5%)	2 (8%)
Upper secondary education	3 (6%)	2 (9%)	1 (4%)
Vocational	16 (34%)	8 (36%)	8 (32%)
Short further ( < 3 y)	2 (4%)	0 (0%)	2 (8%)
Medium further (3-4 y)	15 (32%)	9 (41%)	6 (24%)
Long further ( > 4 y)	8 (17%)	2 (9%)	6 (24%)
Family status, *n* (%):			
Living alone with or without children	18 (38)	10 (45)	8 (32)
Living with a partner with or without children at home	29 (62)	12 (55)	17 (68)
Diabetes duration (years)	16 (8, 20)	16 (12, 20)	12 (7, 19)
HbA1c, mmol/mol	63 (57, 73)	63 (57, 69)	63 (57, 75)
HbA1c, %	7.9 (7.4, 8.8)	7.9 (7.4, 8.5)	7.9 (7.4, 9.0)
Blinded CGM data:			
MAGE, mmol/l	4.3 (3.1, 5.4)	4.4 (3.7, 5.4)	4.0 (2.9, 5.2)
Mean plasma glucose, mmol/l	8.9 (7.5, 9.5)	8.5 (7.3, 9.3)	9.0 (7.6, 10.0)
Glycaemic variability, CV, %	22. 8 (19.2, 28.6)	24.9 (21.2, 29.2)	20.9 (18.1, 27.0)
Glycaemic variability, SD, mmol/l	2.1 (1.6, 2.7)	2.0 (1.8, 2.5)	2.1 (1.6, 2.9)
TIR: % time spent 3.9–10.0 mmol/l	70.1 (54.1, 86.2)	78.0 (63.4, 86.9)	68.5 (49.0, 80.4)
TAR: % time spent 10.1–13.9 mmol/l	22.2 (10.4, 43.5)	18.9 (10.3, 33.4)	27.7 (18.2, 49.1)
TBR: % time spent 3.0–3.8 mmol/l	0.00 (0.0, 0.3)	0.0 (0.0, 0.5)	0.0 (0.0, 0.1)
Body weight, kg	94.5 (86.1, 109.2)	95.7 (86.1, 109.2)	92.3 (86.6, 102.8)
BMI, kg/m^2^	31.0 (27.7, 36.0)	33.3 (27.4, 36.0)	30.5 (28.4, 34.1)
Waist/Hip ratio, unitless (men)	1.08 (1.05, 1.12)	1.08 (1.06, 1.42)	1.09 (1.04, 1.10)
Waist/Hip ratio, unitless (women)	0.96 (0.95, 1.01)	0.97 (0.96, 1.02)	0.96 (0.92, 0.99)
Systolic/diastolic pressure, mmHg	136 (17)/80 (11)	138 (18)/82 (11)	134 (16)/79 (10)
LDL cholesterol, mmol/l	1.7 (1.1, 2.1)	1.7 (1.2, 2.1)	1.6 (1.0, 2.2)
Antihyperglycaemics, *n* (%):			
Metformin	41 (87)	19 (86)	22 (88)
SU	0	0	0
GLP-1RAs	34 (72)	15 (68)	19 (76)
DPP-4s	2 (4)	1 (5)	1 (4)
SGLT-2s	27 (57)	12 (55)	15 (60)
Basal insulin	26 (55)	14 (64)	12 (48)
Prandial insulin	8 (17)	3 (14)	5 (20)
Insulin users, all types	26 (55)	14 (64)	12 (48)
Antihypertensives, *n* (%)	40 (85)	20 (91)	20 (80)
Lipid-lowering medication, *n* (%)	40 (85)	18 (82)	22 (88)

^a^Dropouts before baseline measurements are not included (*N* = 1 in the BCC group). Data are mean (SD) or medians (25th and 75th percentiles). Categorical data will be summarized by numbers and percentages.

*BCC* basic carbohydrate counting, *BMI* body mass index, *CGM* continuous glucose monitoring, *CV* coefficient of variation, *DPP-4s* dipeptidyl peptidase 4 inhibitors, *GLP-1RAs* glucagon-like peptide 1 receptor agonizts, *HbA1c* glycated hemoglobin A1c, *LDL* low-density lipoprotein, *MAGE* mean amplitude of glycaemic excursions, *SGLT-2s* sodium-glucose co-transporter 2 inhibitors, *SD* standard deviation, Standard, standard dietary treatment, *SU* sulfonylureas, *TAR* time above range, *TBR* time below range, *TIR* time in range.

### Primary outcomes

**Fig. 1:**
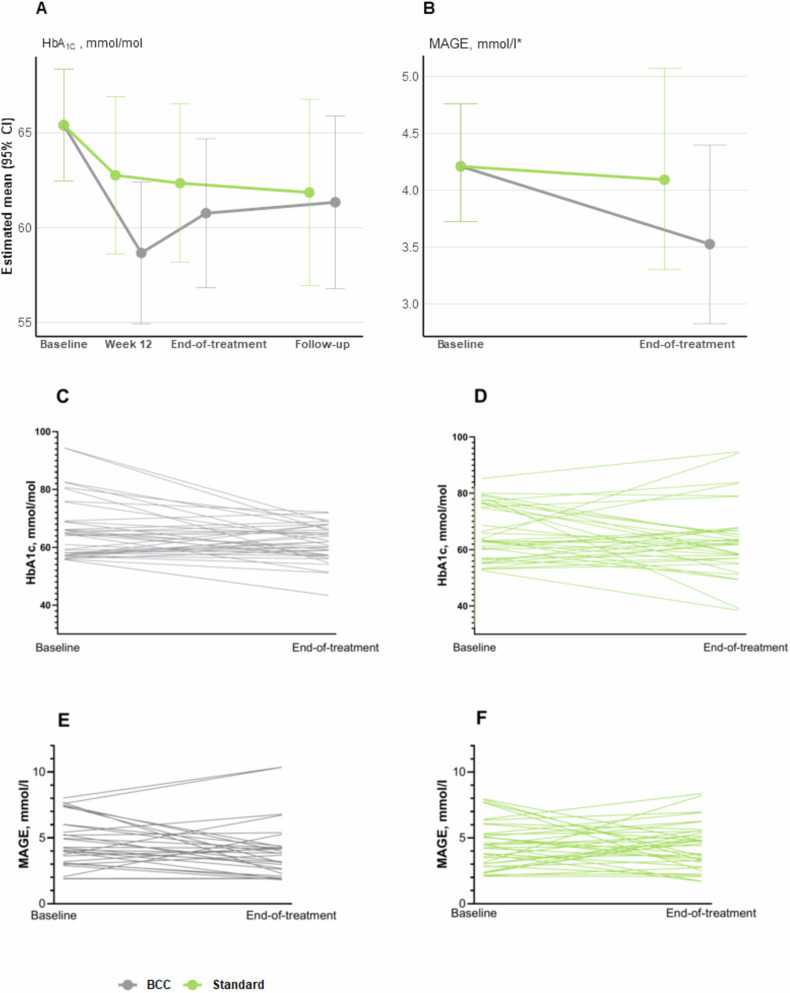
Glycaemic changes. Changes in primary outcomes: **A** HbA1c changes from baseline to end-of-treatment for both groups; **B** MAGE changes from baseline to end-of-treatment for both groups; **C** Spaghetti plot of individual HbA1c changes from baseline to end- of-treatment for completers in the BCC group; **D** Spaghetti plot of individual HbA1c changes from baseline to end- of-treatment for completers in the standard group; **E** Spaghetti plots of individual MAGE changes from baseline to end-of-treatment for completers in the BCC group; **F** Spaghetti plots of individual MAGE changes from baseline to end-of-treatment for completers in the standard group.

Compared with the standard dietary care group, we observed no treatment effects of the BCC intervention on HbA1c (−2 mmol/mol (−7 to 4 [−0.2%, −0.6 to 0.4])); (*p* = 0.554), or MAGE (−14% (−36 to 16); *p* = 0.319) from baseline to end-of-treatment (Fig. 1A, B, Table 2). Both groups experienced comparable reductions in HbA1c and MAGE from baseline to end-of-treatment at 6 months (Table 2). Individual changes in HbA1c and MAGE from baseline to end-of-treatment for completers are presented in Fig. 1C–F.

**Table 2:** Baseline-adjusted estimates for primary and secondary/exploratory outcomes.

Outcome	Group	Visit	Estimated mean (95% CI)	Within-group changes (95% CI)	Difference from Control (95% CI)	*P*-value
**HbA1c, mmol/mol**	BCC	Baseline	65 (62: 68)			
		Week 12	59 (55: 62)	−7 (−10: −3)	−4 (−9: 1)	0.119
		End-of-treatment	61 (57: 65)	−5 (−8: −1)	−2 (−7: 4)	0.554
		Follow-up	61 (57: 66)	−4 (−8: 0)	−1 (−7: 6)	0.865
	Standard	Baseline	65 (62: 68)			
		Week 12	63 (59: 67)	−3 (−7: 1)		
		End-of-treatment	62 (58: 67)	−3 (−7: 1)		
		Follow-up	62 (57: 67)	−4 (−8: 1)		
**HbA1c, %**	BCC	Baseline	8.1 (7.8: 8.4)			
		Week 12	7.5 (7.2: 7.8)	−0.6 (−0.9: −0−3)	−0.4 (−0.8: 0.1)	0.119
		End-of-treatment	7.7 (7.4: 8.1)	−0.5 (−0.7: −0.1)	−0.2 (−0.6: 0.4)	0.554
		Follow-up	7.7 (7.4: 8.2)	−0.4 (−0.7: 0.0)	−0.1 (−0.6: 0.6)	0.865
	Standard	Baseline	8.1 (7.8: 8.4)			
		Week 12	7.9 (7.5: 8.3)	−0.3 (−0.6: 0.1)		
		End-of-treatment	7.8 (7.5: 8.3)	−0.3 (−0.6: 0.1)		
		Follow-up	7.8 (7.4: 8.3)	−0.4 (−0.7: 0.1)		
**MAGE, mmol/l**^a^	BCC	Baseline	4.2 (3.7: 4.8)			
		End-of-treatment	3.5 (2.8: 4.4)	−16 (−33: 5)	−14 (−36: 16)	0.319
	Standard	Baseline	4.2 (3.7: 4.8)			
		End-of-treatment	4.1 (3.3: 5.1)	−3 (−21: 20)		
**Mean plasma glucose, mmol/l**^a^	BCC	Baseline	9.0 (8.5: 9.6)			
		End-of-treatment	8.7 (7.8: 9.6)	−0.3 (−1.3: 0.6)	−0.2 (−1.5: 1.0)	0.684
	Standard	Baseline	9.0 (8.5: 9.6)			
		End-of-treatment	9.0 (8.1: 9.9)	−0.1 (−1.0: 0.8)		
**TIR (3.9–10.0** **mmol/l), % of time spent**	BCC	Baseline	66.0 (58.4: 73.7)			
		End-of-treatment	70.1 (58.6: 81.6)	4.1 (−7.1: 15.2)	−0.3 (−15.5: 14.9)	0.968
	Standard	Baseline	66.0 (58.4: 73.7)			
		End-of-treatment	70.4 (59.0: 81.9)	4.4 (−6.6: 15.4)		
**TAR (10.1−13.9** **mmol/l), % of time spent**	BCC	Baseline	31.2 (23.5: 38.9)			
		End-of-treatment	27.9 (16.4: 39.3)	−3.3 (−14.7: 8.1)	−0.1 (−15.5: 15.4)	0.994
	Standard	Baseline	31.2 (23.5: 38.9)			
		End-of-treatment	27.9 (16.4: 39.4)	−3.3 (−14.5: 8.0)		
**CV for plasma glucose, %**	BCC	Baseline	23.7 (21.7: 25.8)			
		End-of-treatment	25.5 (21.7: 29.3)	1.8 (−1.8: 5.3)	2.9 (−2.0: 7.8)	0.239
	Standard	Baseline	23.7 (21.7: 25.8)			
		End-of-treatment	22.6 (18.9: 26.3)	−1.1 (−4.6: 2.3)		
**BMI, kg/m**^**2**^	BCC	Baseline	32.3 (30.5: 34.0)			
		End-of-treatment	31.7 (29.9: 33.4)	−0.6 (−1.1: −0.1)	−0.3 (−1.0: 0.4)	0.411
		Follow-up	31.9 (30.1: 33.8)	−0.3 (−1.0: 0.4)	0.1 (−1.0: 1.2)	0.851
	Standard	Baseline	32.3 (30.5: 34.0)			
		End-of-treatment	32.0 (30.2: 33.7)	−0.3 (−0.8: 0.2)		
		Follow-up	31.8 (30.0: 33.7)	−0.4 (−1.2: 0.4)		
**Body weight, kg**^a^	BCC	Baseline	96.3 (91.3: 101.5)			
		End-of-treatment	94.5 (89.6: 99.7)	−1.8 (−3.2: −0.4)	−1.2 (−3.3: 1.0)	0.277
		Follow-up	95.2 (89.8: 100.8)	−1.2 (−3.5: 1.2)	−0.2 (−3.6: 3.4)	0.919
	Standard	Baseline	96.3 (91.3: 101.5)			
		End-of-treatment	95.6 (90.6: 100.9)	−0.7 (−2.2: 0.9)		
		Follow-up	95.3 (89.9: 101.0)	−1.0 (−3.5: 1.6)		
**Waist/Hip ratio, unitless**	BCC	Baseline	1.05 (1.03: 1.07)			
		End-of-treatment	1.04 (1.02: 1.07)	−0.01 (−0.03: 0.01)	−0.01 (−0.04: 0.01)	0.310
		Follow-up	1.05 (1.03: 1.08)	0.00 (−0.01: 0.02)	−0.00 (−0.03: 0.03)	0.975
	Standard	Baseline	1.05 (1.03: 1.07)			
		End-of-treatment	1.05 (1.03: 1.08)	0.01 (−0.02: 0.03)		
		Follow-up	1.05 (1.03: 1.08)	0.00 (−0.02: 0.02)		
**Systolic blood pressure, mmHg**	BCC	Baseline	136 (131: 141)			
		End-of-treatment	134 (128: 140)	−2 (−7: 4)	−0 (−8: 7)	0.957
		Follow-up	136 (129: 142)	0 (−6: 6)	1 (−7: 9)	0.764
	Standard	Baseline	136 (131: 141)			
		End-of-treatment	134 (128: 140)	−2 (−7: 4)		
		Follow-up	134 (128: 141)	−1 (−7: 5)		
**Diastolic blood pressure, mmHg**	BCC	Baseline	80 (77: 83)			
		End-of-treatment	78 (74: 82)	−2 (−5: 1)	−1 (−6: 3)	0.591
		Follow-up	81 (77: 84)	0 (−3: 3)	0 (−4: 4)	0.905
	Standard	Baseline	80 (77: 83)			
		End-of-treatment	79 (75: 83)	−1 (−4: 2)		
		Follow-up	80 (77: 84)	−0 (−3: 3)		
**LDL cholesterol, mmol/l**	BCC	Baseline	1.8 (1.5: 2.0)			
		End-of-treatment	1.7 (1.4: 2.1)	−0.0 (−0.3: 0.2)	−0.2 (−0.5: 0.2)	0.284
		Follow-up	1.7 (1.4: 2.0)	−0.1 (−0.4: 0.3)	0.2 (−0.3: 0.6)	0.469
	Standard	Baseline	1.8 (1.5: 2.0)			
		End-of-treatment	1.9 (1.6: 2.3)	0.2 (−0.1: 0.4)		
		Follow-up	1.5 (1.2: 1.9)	−0.2 (−0.6: 0.1)		
**Median carbohydrate estimation errors, g**^a^	BCC	Baseline	67.5 (42.3: 107.7)			
		End-of-treatment	11.6 (6.9: 19.4)	−82.8 (−90.7: −68.5)	−71.0 (−86.1: −39.2)	0.002
		Follow-up	16.2 (8.8: 30.0)	−76.0 (−88.2: −51.0)	−52.8 (−81.7: 22.2)	0.117
	Standard	Baseline	67.5 (42.3: 107.7)			
		End-of-treatment	39.9 (23.4: 68.1)	−40.9 (−68.4: 10.4)		
		Follow-up	34.3 (16.7: 70.4)	−49.2 (−77.1: 13.2)		
**Median carbohydrate estimation errors, %**^a^	BCC	Baseline	139 (95: 203)			
		End-of-treatment	41 (30: 55)	−71 (−80: −58)	−55 (−70: −32)	<0.001^b^
		Follow-up	55 (36: 82)	−61 (−76: −35)	−23 (−58: 41)	0.384
	Standard	Baseline	139 (95: 203)			
		End-of-treatment	90 (65: 124)	−36 (−56: −6)		
		Follow-up	71 (45: 111)	−49 (−70: −14)		

Estimated means (CI 95%) (left) and baseline corrected difference between groups (CI 95%).

^a^Variable has been log-transformed for analysis and back−transformed for presentation, comparisons are presented as relative differences (%). *P*-values are between group differences.

*BCC* basic carbohydrate counting, *BMI* body mass index, *CV* coefficient of variation, *HbA1c* hemoglobin A1c, *LDL* low-density lipoprotein, *MAGE* mean amplitude of glycaemic excursions, Standard, standard dietary treatment, *TAR* time above range, *TBR* time below range, *TIR* time in range.

^b^FDR < 0.05.

### Secondary/exploratory outcomes

**Fig. 2:**
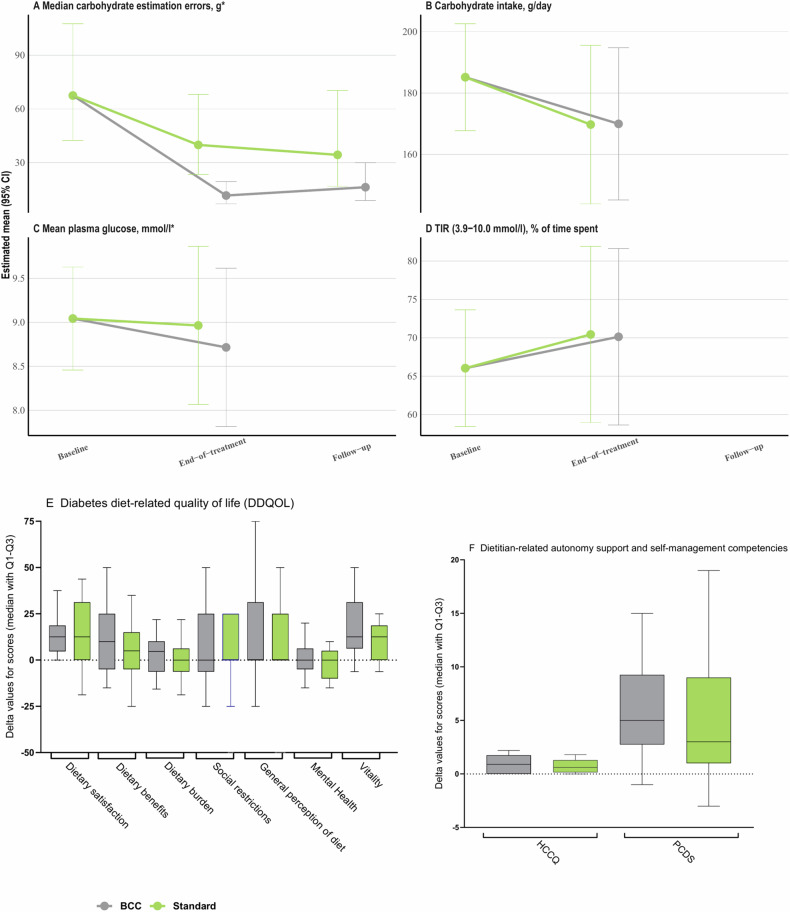
Changes in selected secondary outcomes: **A** Median carbohydrate estimation error, gram; **B** Carbohydrate intake, g/day; **C** Mean plasma glucose (mmol/l); **D** Time in range (TIR), %; **E** Diabetes diet-related quality of life (DDQOL); **F** Perceived dietitian-related autonomy support (HCCQ) and competencies in diet and diabetes (PCDS).

No effects between the groups were found for any of the secondary and exploratory outcomes except for the median carbohydrate estimation error, which changed by −71% (−80 to −58) in the BCC group and by −36% (−56 to −6) in the standard group from a baseline-adjusted mean of 139% (95% CI 95−203) indicating a large overestimation of the carbohydrate content in various high-carbohydrate foods at baseline in both groups. The overall treatment difference was −55% (−70 to −32), *p* < 0.001 at end-of-intervention (Table 2). After adjustment for multiplicity, the treatment difference in median estimation errors in percentage remained significant. Carbohydrate estimation errors at baseline and at end-of-intervention for the individual high-carbohydrate food items included in the test are presented in Fig. S2. Changes from baseline to end-of-intervention in median carbohydrate estimation error, carbohydrate intake, mean plasma glucose, time-in-range, diabetes diet-related quality of life, perceived dietitian-related autonomy support and competencies in diet and diabetes are presented in Fig. 2A–F. Additional supplementary secondary/exploratory outcomes are presented in Tables S5 and S6.

## Discussion

We found that BCC as add-on to standard dietary care did not reduce HbA1c or glucose variability (MAGE) compared with standard dietary care in individuals with T2D. This aligns with findings from previous studies [25, 26]. Interestingly, participants in the BCC group demonstrated notable improvements in accurately estimating carbohydrates across a variety of foods in comparison with the standard care group. This positive outcome can be attributed to the practical educational approach employed, incorporating hands-on exercises for assessing carbohydrate content, portion control calculations, and visualization of commonly consumed serving sizes using real foods. Bowen et al.’s study [25], reinforces our results, highlighting the challenges individuals with T2D, especially those with lower numeracy skills, may face in applying carbohydrate counting efficiently. Other studies have also associated poor literacy and numeracy skills with attenuated skills in accurate portion-size estimation and understanding of nutrition labels [27–30]. A noteworthy finding was the decline in estimation skills observed in both groups at the 6-month follow-up, underscoring the importance of ongoing dietary education and support for maintaining newly acquired skills and competencies. Our results, corroborating previous research [25, 26], challenge the prevailing assumption that elevated carbohydrate counting skills alone can lead to substantial improvements in glycaemic control for individuals with T2D. This underscores the multifaceted nature of diabetes management, where factors beyond carbohydrate counting – including use of antidiabetic medication and other lifestyle modifications – contribute significantly to glycaemic outcomes. Consequently, adopting a more personalized approach becomes paramount. An example of a more refined precision nutrition strategy could be the utilization of continuous glucose monitoring (CGM) as a behavioral tool for instant feedback on the impact of dietary modifications on glycaemic control. While the available evidence to support this approach is currently limited, it warrants additional investigation for its potential efficacy [31, 32].

Despite our efforts, the study faced limitations, including a small sample size and study visit delays due to COVID-19-related disruptions. These factors negatively impacted the statistical power of our trial, meaning that the study results are less reliable with a risk of producing inconclusive results and potentially impacting the ability to detect clinically relevant intervention effects (risk of Type II errors). This may be the reason why the improvements in carbohydrate estimation skills did not have a significant impact on glycaemic control in the BCC group. Changes in medication may also have interfered and affected the results. Nonetheless, our analysis of baseline characteristics revealed that 72% and 88% of participants in the BCC group and the standard dietary care group, respectively, were already prescribed modern glucose-lowering drugs as GLP-1RAs and SGLT-2s prior to study enrollment with a relatively balanced distribution between the two study groups. The slightly higher discontinuation of GLP-1RAs and SGLT-2s in the standard group during the intervention period, could potentially have introduced medication-related interferences in the study outcomes. We adopted a pragmatic study design, acknowledging the inherent probability of adjustments to medication due to clinical or patient-related factors. However, this methodological approach enables the integration of real-world conditions, ultimately augmenting the generalizability of our research findings.

The increased carbohydrate awareness could have led to a reduced carbohydrate consumption. However, we did not find that this was the case in our trial. Total carbohydrate intake remained unchained during the intervention period, and we did not find any other dietary changes. The dietary composition during the study period was very similar to what we have previously found in a comparable Danish population with T2D [33]. The lack of dietary changes may be due to the method used since under-, and misreporting is a well-known bias [34, 35], and has been found highly prevalent in individuals both with previous and current obesity [36, 37] including obese individuals with T2D [38]. Another limitation is the use of MAGE as a primary outcome. Today MAGE is no longer recommended as the standard method for assessing glucose variability as it is inaccurate for measuring glycaemic variability and not designed for use together with CGM. Our study was designed in 2018 and did therefore not include international recommendations such as time in range, time below range, time above range, CV, and SD to assess glycaemic variability [22, 39].

In conclusion, we did not find BCC to improve HbA1c or MAGE when compared with standard care in individuals with longstanding diabetes. Potential reasons for this are the lack of a relevant effect or the small sample size. While participants in the BCC group enhanced their ability to estimate carbohydrate content, indicating at least some degree of effect of the educational program, the lack of a significant impact on glycaemic control prompts further exploration. Our results advocate for a more comprehensive strategy, potentially incorporating real-time CGM for precision nutrition, and underscore the need for larger trials to investigate the potential benefits of combining BCC with CGM in T2D. Lastly, future studies should adopt easily adaptable designs that integrate advanced monitoring technologies alongside educational initiatives and ongoing support in behavioral interventions. This comprehensive approach has the potential to enhance and sustain adherence, contributing to the optimization of long-term outcomes for individuals with T2D, thus fostering the potential sustainability of interventions.

### Supplementary information

Supplementary legends

Supplementary

## Data Availability

The data that support the findings of this study are not openly available due to reasons of sensitivity but are available from the corresponding author upon reasonable request. Data are securely stored within a controlled and restricted access storage system located at Steno Diabetes Center Copenhagen.
