# Supplementary material for: Effects of basic carbohydrate counting versus standard dietary care for glycaemic control in type 2 diabetes (The BCC Study): a randomised, controlled trial
Source: Nutr Diabetes. 2024 Jun 27;14:47. doi: 10.1038/s41387-024-00307-0 (PMC11211433; doi:10.1038/s41387-024-00307-0)
Supplement: Supplementary file 1 — Supplementary legends [file 41387_2024_307_MOESM1_ESM.docx]

**Supplementary legends**

**Appendix A.** Statistical Analyses Plan (SAP)

**Fig. S1** Flow diagram

**Fig. S2** Carbohydrate counting errors at baseline and end-of-treatment at 6 months.

**Table S1** Baseline characteristics supplementary

**Table S2** Number of users of antihyperglycaemics at baseline and end-of-intervention

**Table S3** Number of participants with changes in prescribed dose of antihyperglycaemics at baseline and end-of-intervention

**Table S4** Prescribed antihyperglycaemics, medians (IQR) at baseline and end-of-intervention

**Table S5** Baseline-adjusted estimates for primary and secondary/exploratory outcomes - supplementary

**Table S6** Delta values for diabetes diet-related quality of life, perceived autonomy support and competencies in diet and diabetes
