# Supplementary material for: Effects of basic carbohydrate counting versus standard dietary care for glycaemic control in type 2 diabetes (The BCC Study): a randomised, controlled trial
Source: Nutr Diabetes. 2024 Jun 27;14:47. doi: 10.1038/s41387-024-00307-0 (PMC11211433; doi:10.1038/s41387-024-00307-0)
Supplement: Supplementary file 2 — Supplementary [file 41387_2024_307_MOESM2_ESM.pdf]

Statistical Analysis Plan for the manuscript reporting primary and selected secondary outcomes from the BCC study

Contents

Section 1: Administrative information 3
Signature page 4
Section 2: Introduction 5
Background and Rationale (adapted from study protocol) 5
Objectives 5
Section 3: Study Methods 6
Trial design 6
Randomization 6
Sample size 6
Framework 6
Statistical interim analyses and stopping guidance 6
Timing of final analyses 6
Timing of outcome assessments 6
Section 4: Statistical Principles 7
Confidence intervals and P values 7
Completers and protocol deviations 7
Analysis populations 7
Section 5: Trial Population 8
Screening data 8
Eligibility 8
Recruitment 8
Withdrawal/follow-up 8
Baseline participant characteristics 8
Section 6: Analysis 12
Outcome definitions 12
Analysis methods 15
Missing data 15
Additional analyses 15
Harms 15
Statistical software 15
References 16

Section 1: Administrative information

Title: *Effects of basic carbohydrate counting versus standard outpatient nutritional education: A randomized controlled trial focusing on HbA1c and glucose variability in patients with type 2 diabetes (The BCC Study).*

Publication date: 25 April 2023
SAP version: 1
Protocol version: 8
ClinicalTrials.gov ID: NCT03623139
Journal no.: H-18014918 (The study has been approved by the Danish ethics committee in Copenhagen).

This document is a supplement to the BCC study protocol (1). It contains the statistical analysis plan (SAP) for the main paper of the trial in which the primary outcomes and selected secondary outcomes will be reported. This document complies with the guidelines for content of statistical analysis plans in clinical trials (2).

Principal investigator Bettina Ewers, MSc PhD
Steno Diabetes Center Copenhagen
Borgmester Ib Juuls Vej 83
DK-2730 Herlev
Denmark
E-mail: bettina.ewers@regionh.dk
Phone: +45 3091 2997

Signature page

To be signed by individual writing the Statistical Analysis Plan (SAP), the statistical advisor, contributors to the SAP, principal investigator, and co-investigators.

Title: *Effects of basic carbohydrate counting versus standard outpatient nutritional education: A randomized controlled trial focusing on HbA1c and glucose variability in patients with type 2 diabetes (The BCC Study).*

ClinicalTrials.gov ID: NCT03623139

Name	Title	Role	Signature	Date
Bettina Ewers[1]	MSc, PhD	Principal investigator, data analyst, SAP writer	Bettina Ewers	21.04.23
Martin Bæk Blond[1]	MSc, PhD	Statistical advisor, contributor to SAP	Martin Bæk Blond	240423
Jens Meldgaard Bruun[2,3]	MD, PhD	Co-investigator	[illegible]	23/4 -23
Tina Vilsbøll[1,4]	MD DMSc	Co-investigator	[illegible]	22/4-23

Affiliations

1. Steno Diabetes Center Copenhagen, Herlev, Denmark
2. Steno Diabetes Center Aarhus, Aarhus, Denmark
3. Department of Clinical Medicine, University of Aarhus, Denmark
4. Faculty of Health and Medical Sciences, University of Copenhagen, Denmark

Section 2: Introduction

Background and Rationale (adapted from study protocol)

Carbohydrate is the major energy contributing macronutrient in our diet with the highest impact on plasma glucose levels. Accordingly, the total amount of carbohydrates consumed in a meal is an important predictor of postprandial glucose response (3, 4). The carbohydrate quality (e.g., amounts of dietary fibre or added sugar, and the glycaemic index) also plays a pivotal role in relation to glycaemic response (5). Thus, to control or attenuate postprandial glucose fluctuations, awareness of types and amounts of carbohydrates is of outmost importance. A reduction in postprandial glucose excursions may lead to clinical benefits such as attenuated glucose variability and improvements of glycated haemoglobin A1c (HbA1c) and potentially to a reduction in diabetes-related complications. Carbohydrate counting or similar nutrition educational approaches have been recommended in clinical guidelines for several decades, especially for the management of type 1 diabetes (T1D) (6). However, scientific knowledge of the clinical impact of improving skills in estimating carbohydrate portion sizes and increasing carbohydrate awareness is sparse in people with type 2 diabetes (T2D) (7). Recent studies in people with diabetes suggest that lower literacy and numeracy are associated with poorer portion size estimation skills, poorer understanding of food labels, increased body mass index (BMI), and poorer diabetes-related self-management competencies (8-12). Studies have also found that people with diabetes frequently assess their intake of carbohydrates inaccurately and this has been associated with a higher HbA1c (13-15). Thus, increased knowledge and awareness about carbohydrate-rich foods using a hands-on learning approach for improving numeracy skills and carbohydrate estimation accuracy focusing on a higher within- and between-days consistency in carbohydrate intake may be important to improve glycaemic control in people with T2D.

Objectives

The primary objective of the BCC study is to investigate effects of a nutritional program in basic carbohydrate counting (BCC) as add-on to the usual dietary management on change in HbA1c and glycaemic variability (assessed by mean amplitude of glycaemic excursions (MAGE)) in individuals with T2D.

Secondary objectives were to describe changes associated with the intervention on other clinically relevant metabolic changes (body weight, body composition, blood pressure and lipid profile), changes in skills related to numeracy and carbohydrate estimation accuracy as well as on psychosocial and behavioural outcomes at the end of the intervention period and after 6 months of follow-up to assess long-term maintenance.

<u>Hypotheses for the primary outcomes:</u>

1. We hypothesize that BCC is superior compared with usual dietary care as stand-alone treatment (the control group) in reducing HbA1c or MAGE from baseline to end-of-treatment at week 24 (from V1 to V2).

Superiority is claimed if:

a. the 95% confidence interval for the estimated difference in change between the groups for HbA1c or MAGE, estimated using a baseline corrected linear mixed model, excludes 0 and the P-value is <0.05: and,
b. the estimated difference in HbA1c or MAGE between the two groups is equal to/surpass the minimal important difference in favour of the BCC group.

Hypotheses for the secondary outcomes can be found in the study protocol and clinicaltrials.gov.

Section 3: Study Methods

Trial design

Single-centre parallel-group, randomized controlled, open-label, superiority trial. Allocation ratio 1:1 to either BCC or control (usual dietary care).

Randomization

Participants were randomized using block randomization by stratifying participants based on sex, BMI and HbA1c at baseline. The randomization list was generated by an external statistician and uploaded to the electronic data management system REDCap (8.10.18, Vanderbilt University, TN, USA). Participants eligible for inclusion in the study according to the screening were randomized at the end of the screening visit (V0) by the study investigator/study personnel using the randomization module in REDCap. Baseline measurements were collected at the following baseline visit (V1) for alle participants.

Sample size

See study protocol.

Framework

Superiority trial. See Objectives section.

Statistical interim analyses and stopping guidance

No interim analyses were planned and no guidelines for terminating the trial early was made.

Timing of final analyses

The results will be analysed when this statistical analysis plan has been uploaded at clinicaltrials.gov

Timing of outcome assessments

HbA1c was measured at the screening visit (V0), at the baseline visit (V1), after 12 weeks of intervention, after 24 weeks (V2) of intervention, and after six months follow-up (V3). MAGE was measured after the baseline visit (V1), and after 24 weeks of intervention (after V2).

See section 6: Analyses for timing of secondary and descriptive/explorative outcome assessments.

Section 4: Statistical Principles

Confidence intervals and P values

Two-sided P-values and 95% confidence intervals will be presented for comparisons between groups. Two-sided 95% confidence intervals will be presented for within group comparisons and estimated levels.

Primary outcome: The direction and size of the estimated mean effect for HbA1c and MAGE (primary outcomes), in addition to the 95% confidence intervals, will be required to support the tested hypothesis for the results to be declared in accordance with the hypothesis (see section 2 under "Objectives").

Secondary outcomes: False detection rate (FDR) correction ad modem Benjamini and Hochberg (16) will be used to control for multiplicity; < 5% will be used as the threshold for FDR.

Completers and protocol deviations

Completers: Participants who participated in an assessment of at least one of the primary outcomes at V2 and/or V3.

Lost to follow-up: Participants who did not participate in assessment of primary outcomes at V2.

Excluded for statistical data analyses: Participants in both study groups dropping out or lost to follow-up before baseline measurements have been collected.

Protocol deviators: None of the participants in the two study groups are considered protocol deviators.

Analysis populations

Efficacy estimates based on intention-to-treat (ITT) analysis set:
All participants will be analysed as randomised.

Section 5: Trial Population

Screening data

The following data obtained at the screening visit will be included for those that entered the trial:

- Age
- Self-reported sex
- Self-reported ethnicity
- Self-reported educational level
- Self-reported smoking status and number of years of smoking
- Diabetes duration

Eligibility

- Age: ≥18 to ≤75 years
- Diabetes duration: ≥12 months
- HbA1c: 53–97 mmol/mol
- Diet or any glucose-lowering medication
- Diagnosed with T2D and treated at Steno Diabetes Center Copenhagen

Recruitment

The flow diagram of the trial will comply with the most recent CONSORT guidelines for Reporting Outcomes in Trial Reports and includes:

1. Total number assessed for eligibility
2. Total number excluded (numbers not meeting criteria, declined to participate, other reasons)
3. Total numbers randomized
4. Total number of participants who were randomly allocated to each group
5. The numbers of participants who received and did not receive the allocated treatment in each group
6. The numbers who were analysed for the primary outcomes in each group
8. For each group, losses, and exclusions after randomisation, together with reasons are reported

Withdrawal/follow-up

The level of consent and consent withdrawal will be tabulated. Participants without HbA1c and MAGE measurements at V2 will be regarded as lost-to-follow-up in relation to the primary outcomes during the intervention period and those with a missing HbA1c measurement at V3 will be regarded as lost-to-follow-up in relation to the primary outcome during the follow-up period. The number of participants lost-to-follow-up for each group during each phase of the trial will be reported in the CONSORT diagram. Summary of baseline levels for variables reported in the baseline table will be provided for completers and for those lost to follow-up/discontinuation of intervention after the baseline visit.

Baseline participant characteristics

The distribution of all continuous outcomes included in baseline characteristics will be visually inspected using QQ-plots and histograms; those with a Gaussian distribution will be presented as means and standard deviations and those with a non-Gaussian distribution will be presented as medians plus 25th and 75th percentiles, number of observations will be presented for each outcome presented. Categorical data will be summarised by numbers and percentages. Tests of statistical significance will not be undertaken for baseline

characteristics according to the CONSORT Statement for reporting clinical trials; rather the clinical importance of any imbalance will be noted (17).

The following outcomes will be included in the baseline participant characteristics table for all participants combined and stratified by randomisation group:

- Number of participants, n
- Age (years)
- Gender, male, n and %
- Ethnicity, white, n and %
- Smoking status
    - Current smoker, n and %
    - Previous smoker, n and %
- Number of smoking years
- Education
    - Elementary school, n and %
    - Upper secondary education, n and %
    - Vocational, n and %
    - Short further (< 3 y), n and %
    - Medium further (3-4 y), n and %
    - Long further (> 4 y), n and %
    - Other education, n and %
- Occupation
    - Employed/self-employed, n and %
    - Unemployed / job seeking, n and %
    - Retired, n and %
    - Other (on leave / studying), n and %
- Family status
    - Living alone, n and %
    - Living alone with children at home, n and %
    - Living with a partner and children at home, n and %
    - Living with a partner (no children at home), n and %
    - Other, n and %
- Annual household income (before tax)
    - < 100,000 DKK (<13,500 EUR)
    - 100,000 – 200,000 DKK (13,500 – 27,000 EUR)
    - 200,000 – 400,000 DKK (27,000 – 54,000 EUR)
    - 400,000 – 600,000 DKK (54,000 – 81,000 EUR)
    - 600,000 – 800,000 DKK (81,000 – 108,000 EUR)
    - 800,000 DKK (>108,000 EUR)
    - Unspecified

- Diabetes duration (years)

- Glucose-lowering medication
    - Basal insulin, n and %
    - Prandial insulin, n and %
    - Mixed insulin, n and %
    - Metformin, n and %

  - SU, n and %
  - GLP-1, n and %
  - DPP-4, n and %
  - SGLT2, n and %

- Antihypertensives, n and %
- Lipid-lowering medication, n and %

- Body weight (kg), female
- Body weight (kg), male

- BMI, kg/m^2

- Waist circumference, cm (women)
- Waist circumference, cm (men)
- Waist/Hip ratio (women)
- Waist/Hip ratio (men)

- Energy intake, kJ/day (women)
- Energy intake, kJ/day (men)

- Carbohydrate intake (g/day and percentage of total energy intake (E%)) (women)
- Carbohydrate intake (g/day and E%) (men)

- Physical activity level
  - Low, n and %
  - Moderate, n and %
  - High, n and %

- Systolic blood pressure (mmHg)
- Diastolic blood pressure (mmHg)

- HbA1c (mmol/mol)

- Blinded CGM data
  - MAGE (mmol/l)
  - Mean plasma glucose, mmol/l
  - Time in range (TIR): % of readings and time 3.9-10.0 mmol/l
  - Time above range (TAR): % and readings and time 10.1-13.9 mmol/l
  - Time below range (TBR): % readings and time 3.0-3.8 mmol/l
  - Glycaemic variability, coefficient of variation (CV), %
  - Glycaemic variability, standard deviation (SD), mmol/l

- Fasting concentration of plasma lipids
  - Total cholesterol (mmol/l)
  - LDL cholesterol (mmol/l)
  - HDL cholesterol (mmol/l)

- VLDL cholesterol (mmol/l)
- Triglycerides (mmol/l)

Section 6: Analysis

Outcome definitions

<u>Primary outcomes</u>

HbA1c and MAGE. The treatment effect will be given as the baseline corrected difference in mmol/mol for HbA1c and mmol/l for MAGE between the groups at V2.

<u>Estimation of minimal important difference for primary outcome</u>: Reductions in HbA1c of 3 mmol/mol and 0.3 mmol/l of MAGE were defined as minimal important differences in this trial. This was primarily based on findings from clinical trials of different dietary approaches for reducing HbA1c in T2D (18, 19). Since no studies had examined the effect of dietary interventions using MAGE as an outcome in our target population with T2D at the time. Thus, based on previous findings (18, 19) we expected to find HbA1c reductions around 3 mmol/mol compared to standard care. This was considered clinically relevant as part of a multidisciplinary approach for the management of hyperglycaemia in T2D.

<u>Secondary/descriptive/exploratory outcomes</u>

The treatment effect will be given as the baseline corrected difference between the groups at V2 and V3. No minimal clinically relevant differences were defined for these outcomes.

- HbA1c (mmol/mol) (only for V3)
- Total body weight (kg), women
- Total body weight (kg), men
- BMI (kg/m2)
- Blood pressure
    - Systolic blood pressure (mmHg)
    - Diastolic blood pressure (mmHg)
- Fasting concentration of plasma lipids
    - Total cholesterol (mmol/l)
    - LDL cholesterol (mmol/l)
    - HDL cholesterol (mmol/l)
- Anthropometry
    - Waist circumference, cm (women)
    - Waist circumference, cm (men)
    - Waist/Hip ratio, unitless (women)
    - Waist/Hip ratio, unitless (men)
- Blinded CGM data (not measured at V3)
    - Mean plasma glucose, mmol/l
    - Time in range (TIR): % of readings and time 3.9-10.0 mmol/l
    - Time above range (TAR): % and readings and time 10.1-13.9 mmol/l
    - Time below range (TBR): % readings and time 3.0-3.8 mmol/l
    - Glycaemic variability, coefficient of variation (CV), %
    - Glycaemic variability, standard deviation (SD), mmol/l

- Numeracy skills test

- Total score
- Correct answers, n and %

- Carbohydrate estimation accuracy test based on 11 high-carb foods (only for foods reported eaten)
  - Estimation errors in %

- Self-reported diabetes diet-related quality of life (DDRQOL), total score within each subscale
  - General perception of diet
  - Satisfaction with diet
  - Restriction of social functions
  - Burden of dietary therapy
  - Perceived benefits of dietary therapy
  - Mental health
  - Vitality

- Self-reported Perceived Health Competence Scale (PCS), total score

- Self-reported Healthcare Climate Questionnaire (HCCQ) (not measured at V3), total score

- Dietary intake (not measured at V3)
  - Total energy intake (kJ/day)
  - Carbohydrate intake (E%)
  - Total fat (E%)
  - Protein (E%)
  - Alcohol (E% and units/day) (women)
  - Alcohol (E% and units/day) (men)
  - Saturated fat (g/day) (women)
  - Saturated fat (g/day) (men)
  - Monounsaturated fat (g/day) (women)
  - Monounsaturated fat (g/day) (men)
  - Polyunsaturated fat (g/day) (women)
  - Polyunsaturated fat (g/day) (men)
  - Dietary fibre (g/day and g/10 MJ) (women)
  - Dietary fibre (g/day and g/10 MJ) (men)
  - Added sugar (g/day and g/10 MJ) (women)
  - Added sugar (g/day and g/10 MJ) (men)

- Self-reported physical activity (International Physical Activity Questionnaire – Short Form (IPAQ SF))
  - MET-minutes/week
  - Low, n and %
  - Moderate, n and %
  - High, n and %

<u>Other outcomes</u>
Number of visits with dietitians, endocrinologists, and diabetes nurses at V2 and V3 in each group.

Supplementary table with baseline characteristics for completers vs. non-completers with dropout before V2
- Number of participants, n
- Age (years)

- Gender, male, n and %
- Ethnicity, white, n and %
- Current smokers, n and %
- Self-reported educational level
    - Elementary school, n and %
    - Upper secondary education, n and %
    - Vocational, n and %
    - Short further (< 3 y), n and %
    - Medium further (3-4 y), n and %
    - Long further (> 4 y), n and %
    - Other education, n and %
- Living alone, n and %
- Body weight (kg), female
- Body weight (kg), male
- BMI, kg/m^2
- Diabetes duration (years)
- HbA1c (mmol/l)

Analysis methods

Analyses of the primary outcomes will be performed based on efficacy estimates.

Adjustment for relevant confounders will be performed including adjustment for design variables used for stratified randomization based on the participants' gender, BMI and HbA1c at baseline.

Before further analysis and before unblinding, all variables will be inspected to detect outliers to uncover potential errors, such as registration errors.

All continuous outcomes covered by this SAP will as a rule be modelled using baseline corrected repeated measures regression (20) with the following fixed effects and interactions between fixed effects: Visit, Visit (factorial)*Treatment. Data from V1, V2, and V3 will be included in the analysis. The models will be specified with a restricted maximum likelihood estimation method and a repeat on participant level (unstructured covariance structure). Model fit will be evaluated using graphical methods before estimating the treatment effects and if necessary, outcomes will be log-transformed. Estimated mean differences (CI95%) between groups (CI95%), conditional means (CI95%), and within group changes (CI95%) will be extracted from the model. For log-transformed outcomes the results will be back-transformed and be presented as the ratio between estimated mean differences (CI95%), estimated conditional geometric means (CI95%) and relative changes within groups (CI95%), respectively. If distribution assumptions cannot be met by log-transformation, a generalized mixed linear model with an appropriate distribution will be applied instead of the repeated measures regression model. In case the distribution does not comply with the distributions available in the generalized mixed linear model a non-parametric test will be used to compare the change scores for the given outcome.

Missing data

The number/frequency of missing values for the primary outcomes in each group at each time point will be provided. In the primary analysis, missing data are handled implicitly by maximum likelihood estimation in the linear mixed model and missing data will be assumed to be missing at random. This is equivalent to making multiple imputations for each treatment group separately and estimating the treatment effect that would have been found had all subject completed their assigned treatment (efficacy estimate) under the missing at random assumption.

Additional analyses

Not relevant.

Harms

Data on harms are not systematically collected and will not be reported.

Statistical software

SAS Enterprise Guide software version 8.3 or newer (SAS Institute Inc., Cary, NC, USA) and R software version 4.0.2 or newer (R Core Team, R Foundation for Statistical Computing, Vienna, Austria.

References

1. Ewers B, Bruun JM, Vilsbøll T. Effects of basic carbohydrate counting versus standard outpatient nutritional education (The BCC Study): study protocol for a randomised, parallel open-label, intervention study focusing on HbA1c and glucose variability in patients with type 2 diabetes. BMJ Open 2019;9:e032893. doi:10.1136/bmjopen-2019-032893
2. Gamble C, Krishan A, Stocken D, Lewis S, Juszczak E, Doré C et al. Guidelines for the content of statistical analysis plans in clinical trials. JAMA 2017; 318: 2337–2343
3. Sheard NF, Clark NG, Brand-Miller JC, Franz MJ, Pi-Sunyer FX, Mayer-Davis E, et al. Dietary carbohydrate (amount and type) in the prevention and management of diabetes: a statement by the American Diabetes Association. Diabetes Care. 2004; 27:9:2266-71.
4. Korsmo-Haugen KK, Brurberg KG, Mann J, Aas AA. Carbohydrate quantity in the dietary management of type 2 diabetes: A systematic review and meta-analysis. Diabetes Obes Metab. 2019; 21:15-27.
5. Evert AB, Dennison M, Gardner CD et al. Nutrition Therapy for Adults with Diabetes or Prediabetes: A Consensus Report. Diabetes Care 2019; 42:5:731-754
6. Franz MJ, MacLeod J, Evert A et al. Academy of Nutrition and Dietetics Nutrition Practice Guideline for Type 1 and Type 2 Diabetes in Adults: Systematic Review of Evidence for Medical Nutrition Therapy Effectiveness and Recommendations for Integration into the Nutrition Care Process. Journal of the Academy of Nutrition and Dietetics 2017; 117:10:1659-1679.
7. Bowen ME, Cavanaugh KL, Wolff K, Davis D, Gregory RP, Shintani A, et al. The diabetes nutrition education study randomized controlled trial: A comparative effectiveness study of approaches to nutrition in diabetes self-management education. Patient Educ Couns. 2016; 99:8:1368-76.
8. Bowen ME, Cavanaugh KL, Wolff K, Davis D, Gregory B, Rothman RL. Numeracy and dietary intake in patients with type 2 diabetes. Diabetes Educ. 2013; 39:2:240-7.
9. Huizinga MM, Beech BM, Cavanaugh KL, Elasy TA, Rothman RL. Low numeracy skills are associated with higher BMI. Obesity (Silver Spring). 2008; 16:8:1966-8.
10. Huizinga MM, Carlisle AJ, Cavanaugh KL, Davis DL, Gregory RP, Schlundt DG, et al. Literacy, numeracy, and portion-size estimation skills. Am J Prev Med. 2009; 36: 4:324-8.
11. Rothman RL, DeWalt DA, Malone R, Bryant B, Shintani A, Crigler B, et al. Influence of patient literacy on the effectiveness of a primary care-based diabetes disease management program. JAMA 2004; 292:14: 1711-6.
12. Rothman RL, Housam R, Weiss H, Davis D, Gregory R, Gebretsadik T, et al. Patient understanding of food labels: the role of literacy and numeracy. Am J Prev Med. 2006; 31:5::391-8.
13. Bishop FK, Maahs DM, Spiegel G, Owen D, Klingensmith GJ, Bortsov A, et al. The Carbohydrate Counting in Adolescents with Type 1 Diabetes (CCAT) Study. Diabetes Spectrum. 2009; 22:1:56-62.
14. Mehta SN, Quinn N, Volkening LK, Laffel LM. Impact of carbohydrate counting on glycemic control in children with type 1 diabetes. Diabetes Care. 2009; 32:6:1014-6.
15. Smart CE, Ross K, Edge JA, King BR, McElduff P, Collins CE. Can children with Type 1 diabetes and their caregivers estimate the carbohydrate content of meals and snacks? Diabet Med. 2010;27(3):348-53.
16. Benjamini Y, Hochberg Y. Benjamini Y, Hochberg Y. Controlling the false discovery rate: a practical and powerful approach to multiple testing. J R Stat Soc B 1995; 57: 289–300.
17. Butcher NJ, Monsour A, Mew EJ. Guidelines for Reporting Outcomes in Trial Reports The CONSORT-Outcomes 2022 Extension. Clinical Review and Education. JAMA Special Communication. JAMA 2022: 328:22:2252-2264
18. Schwingshackl L, Chaimani A, Hoffmann G, Schwedhelm C, Boeing H. A network meta-analysis on the comparative efficacy of different dietary approaches on glycaemic control in patients with type 2 diabetes mellitus. Eur J Epidemiol 2018: 33(2):157– 170. https://doi.org/10.1007/s10654-017-0352-x(012 3456789

19. Long-term Effects of a Lifestyle Intervention on Weight and Cardiovascular Risk Factors in Individuals with Type 2 Diabetes Mellitus Four-Year Results of the Look AHEAD Trial. The Look AHEAD Research Group. Arch Intern Med. 2010; 170(17): 1566–1575. doi:10.1001/archinternmed.2010.334
20. Liu GF, Lu K, Mogg R, Mallick M, Mehrotra D V. Should baseline be a covariate or dependent variable in analyses of change from baseline in clinical trials? Stat Med 2009; 28: 2509-2530.

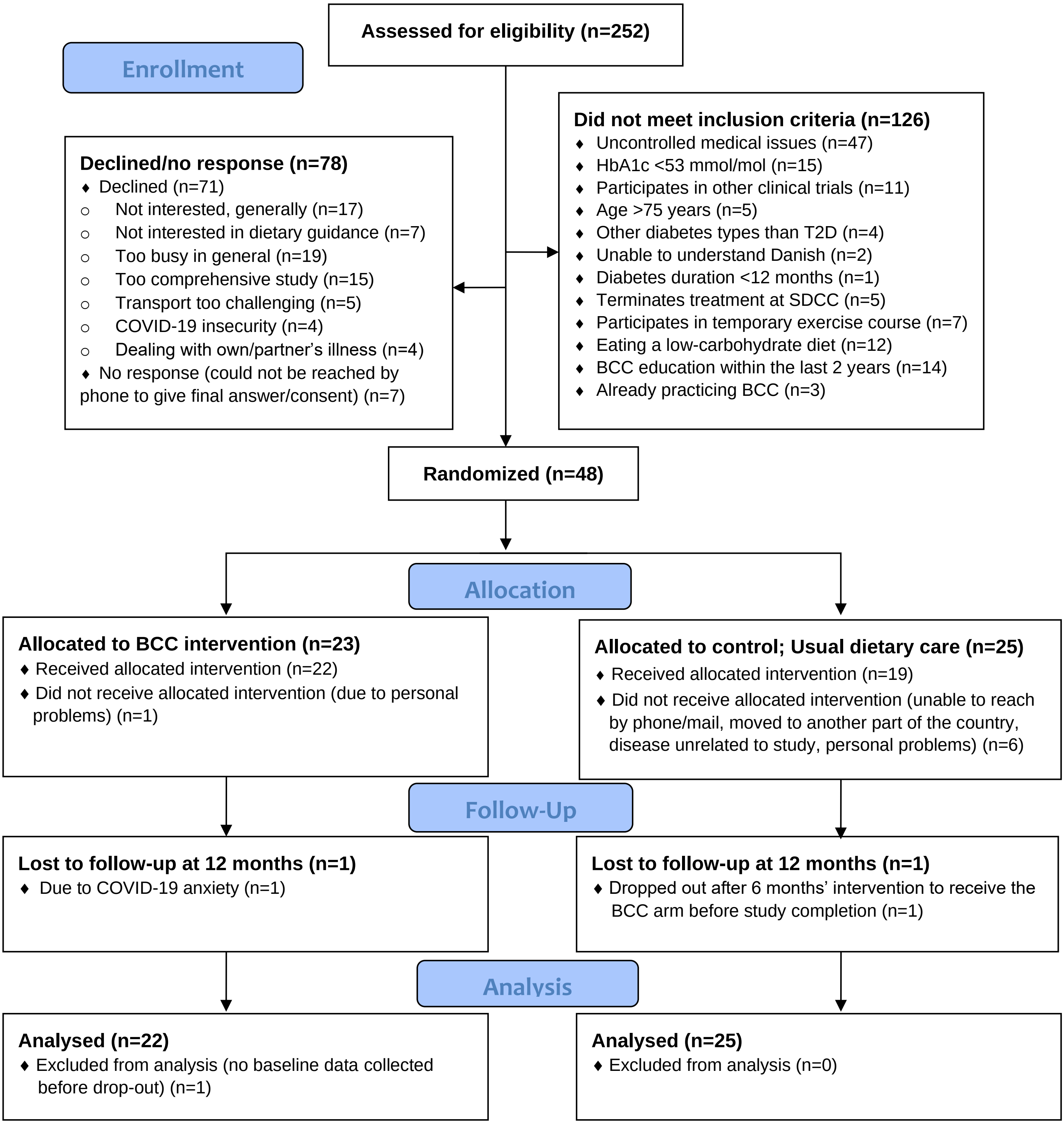

Fig. S1 Flow diagram

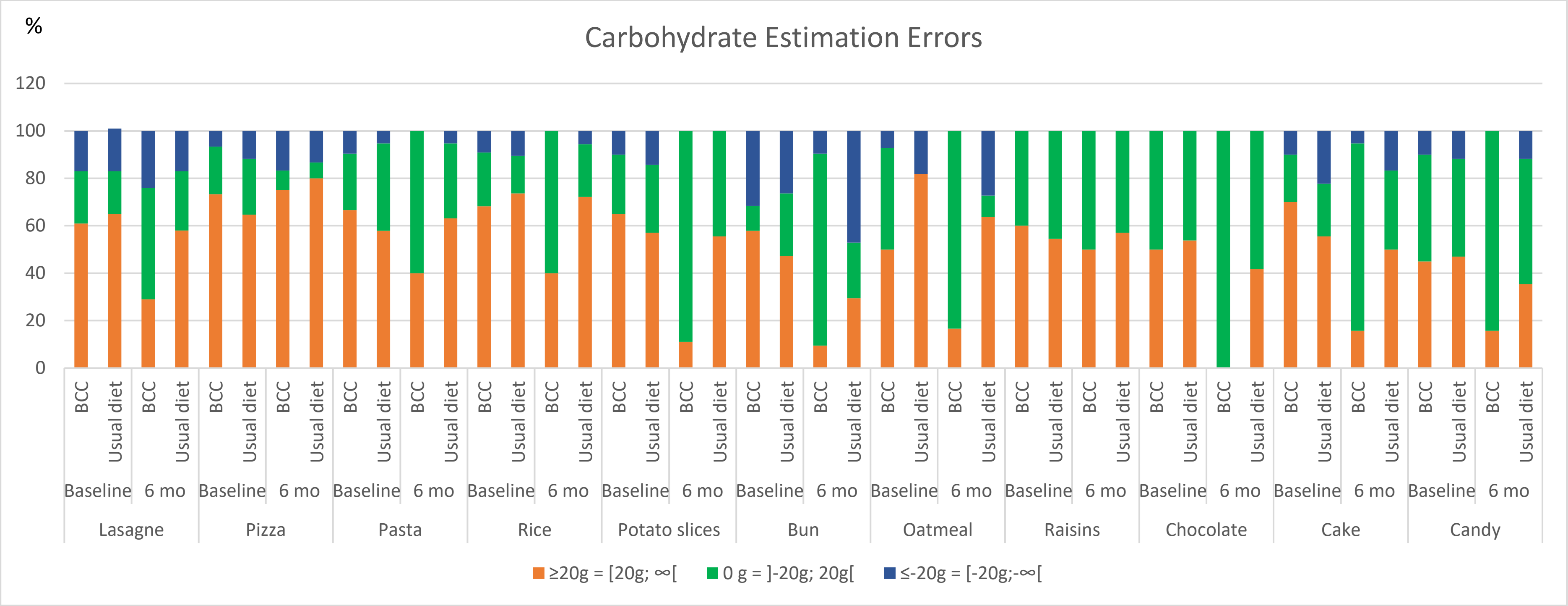

Fig. S2 Carbohydrate counting estimation errors at baseline, and end-of-intervention at 6 months. Distribution of carbohydrate estimation errors above 20-gram errors (red); between 0- and 20-gram errors (green); and below 20-gram errors (blue) for 11 commonly eaten high-carbohydrate foods presented for the two study groups. Only participants who answered that they ate the food at least in between were asked to assess the carbohydrate content in the presented food item. Abbreviations: BCC, basic carbohydrate counting; mo, months.

Table S1 – Baseline characteristics supplementary

**Characteristics**	**Overall** (n=47)	**BCC** (n=22)	**STANDARD** (n=25)
Occupation, n (%):			
Employed/self-employed	26 (55)	12 (55)	14 (56)
Unemployed/job seeking	1 (2)	1 (5)	-
Retired and other*	20 (42)	9 (41)	11 (44)
Annual household income (before tax):			
< 100,000 DKK (<13,500 EUR)	1 (2)	1 (5)	-
100,000 – 200,000 DKK (13,500 – 27,000 EUR)	5 (11)	1 (5)	4 (16)
200,000 – 400,000 DKK (27,000 – 54,000 EUR)	11 (23)	5 (23)	6 (24)
400,000 – 600,000 DKK (54,000 – 81,000 EUR)	11 (23)	7 (32)	4 (16)
600,000 – 800,000 DKK (81,000 – 108,000 EUR)	5 (11)	1 (5)	4 (16)
800,000 DKK (>108,000 EUR)	13 (28)	6 (27)	7 (28)
Unspecified	1 (2)	1 (5)	-
Waist circumference, cm (men)	108 (100, 120)	112 (102, 123)	107 (99, 116)
Waist circumference, cm (women)	116 (104, 123)	121 (104, 124)	111 (101, 118)
Fasting plasma lipids:			
Total cholesterol, mmol/l	3.6 (3.1, 4.3)	3.9 (3.3, 4.3)	3.5 (3.1, 4.2)
HDL cholesterol, mmol/l	1.1 (1.0, 1.3)	1.2 (0.9, 1.4)	1.1 (1.0, 1.2)
Triglycerides, mmol/l	1.8 (1.2, 2.5)	1.8 (1.2, 2.6)	1.8 (1.2, 2.3)
Dietary intake:			
Total energy, kJ/day (men)	8495 (6835, 9970)	7069 (6456, 9301)	8818 (7664, 9990)
Total energy, kJ/day (women)	6202 (5188, 9028)	6046 (4254, 9139)	6359 (5188, 9028)
Carbohydrates, g/day (men)	201 (148, 234)	190 (148, 229)	212 (153, 249)
Carbohydrates, g/day (women)	159 (126, 216)	164 (97, 216)	154 (126, 234)
Carbohydrates, E%	43 (39,46)	45 (39, 47)	41 (39, 45)
Physical activity level, n (%):			
Low	18 (38)	8 (36)	10 (40)
Moderate	17 (36)	8 (36)	9 (36)
High	12 (26)	6 (27)	6 (24)

*Other defined as on leave/studying. Abbreviations: E%, percentage of total energy intake; HDL, high-density lipoprotein.

Table S2 – Number of users of antihyperglycaemics at baseline and end-of-intervention

	**BCC** (n=22)		**STANDARD** (n=25)	
**Antihyperglycaemics**	**Baseline**	**End-of-treatment**	**Baseline**	**End-of-treatment**
Metformin, n	19	19	22	18
SU, n	0	0	0	0
GLP-1Ras, n	15	15	19	14
DPP-4s, n	1	1	1	1
SGLT-2s, n	12	13	15	11
Basal insulin, n	14	14	12	10
Prandial insulin, n	3	3	5	5

DPP-4s, dipeptidyl peptidase 4 inhibitors; GLP-1RAs, glucagon-like peptide 1 receptor agonists; SGLT-2s, sodium-glucose co-transporter 2 inhibitors; SU, sulfonylureas.

Table S3 – Number of participants with changes in prescribed dose of antihyperglycaemics at baseline and end-of-intervention

**Antihyperglycaemics**	**BCC** (n=22)	**STANDARD** (n=25)
Metformin, n	0	4
SU, n	0	0
GLP-1Ras, n	6	14
DPP-4s, n	0	0
SGLT-2s, n	4	6
Basal insulin, n	9	9
Prandial insulin, n	2	4

DPP-4s, dipeptidyl peptidase 4 inhibitors; GLP-1RAs, glucagon-like peptide 1 receptor agonists; SGLT-2s, sodium-glucose co-transporter 2 inhibitors; SU, sulfonylureas.

Table S4 – Prescribed antihyperglycaemics, medians (IQRs) at baseline and end-of-intervention

	**BCC** (n=22)		**STANDARD** (n=25)	
**Antihyperglycaemics**	**Baseline**	**End-of-treatment**	**Baseline**	**End-of-treatment**
Metformin, mg/day	2000 (2000, 2000)	2000 (2000, 2000)	2000 (1000, 2000)	1500 (1000, 2000)
SU, mg/day	-	-	-	-
GLP-1Ras, mg/d	1.0 (1.0, 1.0)	1.0 (1.0, 1.6)	1.0 (0.5, 1.0)	1.0 (1.0, 1.0)
DPP-4s, mg/day	5 (5,5)	5 (5,5)	5 (5,5)	5 (5,5)
SGLT-2s, mg/day	25 (25, 25)	25 (25, 25)	25 (18, 25)	25 (10, 25)
Basal insulin, units/day	52 (32,77)	63 (39,70)	36 (25, 72)	50 (28, 86)
Prandial insulin, units/day	25 (20,36)	18 (17,30)	17 (14, 32)	31 (30, 42)

DPP-4s, dipeptidyl peptidase 4 inhibitors; GLP-1RAs, glucagon-like peptide 1 receptor agonists; SGLT-2s, sodium-glucose co-transporter 2 inhibitors; SU, sulfonylureas.

Table S5 Baseline-adjusted estimates for primary and secondary/exploratory outcomes - supplementary

Outcome	Group	Visit	Estimated mean (95% CI)	Within-group changes (95% CI)	Difference from Control (95% CI)	P-value
**TBR (3.0-3.8 mmol/l), % of time spent**	BCC	Baseline	1.4 (0.3: 2.5)			
		End-of-treatment	1.0 (0.1: 1.9)	-0.4 (-1.8: 1.0)	0.1 (-1.2: 1.5)	0.831
	Standard	Baseline	1.4 (0.3: 2.5)			
		End-of-treatment	0.9 (-0.1: 1.8)	-0.5 (-1.9: 0.8)		
**SD of mean plasma glucose, mmol/L***	BCC	Baseline	2.2 (1.9: 2.4)			
		End-of-treatment	2.3 (1.8: 2.7)	0.1 (-0.3: 0.5)	0.3 (-0.3: 0.9)	0.271
	Standard	Baseline	2.2 (1.9: 2.4)			
		End-of-treatment	1.9 (1.5: 2.4)	-0.2 (-0.6: 0.2)		
**Average waist, cm**	BCC	Baseline	111 (107: 115)			
		End-of-treatment	109 (106: 113)	-2 (-3: 0)	-1 (-4: 1)	0.332
		Follow-up	111 (107: 115)	-0 (-2: 2)	0 (-3: 3)	0.866
	Standard	Baseline	111 (107: 115)			
		End-of-treatment	111 (107: 115)	-0 (-2: 1)		
		Follow-up	111 (107: 115)	-0 (-2: 2)		
**Total cholesterol, mmol/l**	BCC	Baseline	3.8 (3.5: 4.1)			
		End-of-treatment	3.7 (3.4: 4.1)	-0.1 (-0.3: 0.2)	-0.2 (-0.6: 0.1)	0.220
		Follow-up	3.8 (3.4: 4.1)	-0.0 (-0.4: 0.3)	0.3 (-0.2: 0.7)	0.299
	Standard	Baseline	3.8 (3.5: 4.1)			
		End-of-treatment	4.0 (3.6: 4.3)	0.2 (-0.1: 0.4)		
		Follow-up	3.5 (3.1: 3.9)	-0.3 (-0.7: 0.1)		
**HDL cholesterol, mmol/l**	BCC	Baseline	1.14 (1.04: 1.24)			

Table S5 Baseline-adjusted estimates for primary and secondary/exploratory outcomes - supplementary

Outcome	Group	Visit	Estimated mean (95% CI)	Within-group changes (95% CI)	Difference from Control (95% CI)	P-value
		End-of-treatment	1.24 (1.12: 1.36)	0.10 (0.04: 0.16)	0.05 (-0.03: 0.14)	0.221
		Follow-up	1.21 (1.10: 1.32)	0.07 (0.00: 0.13)	0.03 (-0.06: 0.13)	0.518
	Standard	Baseline	1.14 (1.04: 1.24)			
		End-of-treatment	1.19 (1.07: 1.31)	0.05 (-0.02: 0.11)		
		Follow-up	1.18 (1.06: 1.29)	0.04 (-0.03: 0.11)		
**Triglyceride, mmol/l***	BCC	Baseline	1.91 (1.57: 2.32)			
		End-of-treatment	1.61 (1.33: 1.94)	-15.63 (-27.11: -2.34)	-12.69 (-28.55: 6.70)	0.179
		Follow-up	1.79 (1.47: 2.19)	-5.82 (-19.31: 9.94)	13.72 (-8.47: 41.30)	0.238
	Standard	Baseline	1.91 (1.57: 2.32)			
		End-of-treatment	1.84 (1.51: 2.24)	-3.37 (-17.47: 13.16)		
		Follow-up	1.58 (1.28: 1.95)	-17.18 (-29.98: -2.04)		
**Numeracy skills test, correct answers, %**	BCC	Baseline	53 (45: 61)			
		End-of-treatment	57 (47: 67)	4 (-4: 12)	3 (-9: 14)	0.656
		Follow-up	54 (42: 65)	0 (-9: 9)	2 (-11: 15)	0.759
	Standard	Baseline	53 (45: 61)			
		End-of-treatment	54 (44: 65)	1 (-8: 10)		
		Follow-up	52 (40: 63)	-2 (-11: 8)		
**Total energy intake, kJ/day**	BCC	Baseline	7803 (7126: 8480)			
		End-of-treatment	7147 (6258: 8035)	-657 (-1418: 105)	-105 (-1195: 985)	0.846
	Standard	Baseline	7803 (7126: 8480)			

Table S5 Baseline-adjusted estimates for primary and secondary/exploratory outcomes - supplementary

Outcome	Group	Visit	Estimated mean (95% CI)	Within-group changes (95% CI)	Difference from Control (95% CI)	P-value
		End-of-treatment	7252 (6331: 8172)	-551 (-1351: 248)		
**Carbohydrate intake, E%**	BCC	Baseline	43 (41: 45)			
		End-of-treatment	43 (40: 46)	1 (-2: 3)	2 (-2: 6)	0.402
	Standard	Baseline	43 (41: 45)			
		End-of-treatment	42 (39: 45)	-1 (-4: 2)		
**Carbohydrate intake, g/day**	BCC	Baseline	185 (168: 203)			
		End-of-treatment	170 (145: 195)	-15 (-38: 8)	0 (-32: 33)	0.988
	Standard	Baseline	185 (168: 203)			
		End-of-treatment	170 (144: 196)	-15 (-39: 9)		
**Total fat, E%**	BCC	Baseline	37 (35: 38)			
		End-of-treatment	36 (33: 39)	-1 (-4: 2)	-0 (-4: 4)	0.953
	Standard	Baseline	37 (35: 38)			
		End-of-treatment	36 (33: 39)	-1 (-4: 2)		
**Protein, E%**	BCC	Baseline	19 (18: 20)			
		End-of-treatment	19 (17: 21)	0 (-2: 2)	-1 (-4: 2)	0.381
	Standard	Baseline	19 (18: 20)			
		End-of-treatment	20 (18: 22)	1 (-1: 4)		
**Saturated fat intake, g/day***	BCC	Baseline	26 (22: 29)			
		End-of-treatment	23 (19: 28)	-10 (-25: 7)	-1 (-23: 27)	0.921
	Standard	Baseline	26 (22: 29)			
		End-of-treatment	23 (19: 29)	-9 (-25: 9)		
**Monounsaturated fat intake, g/day***	BCC	Baseline	19 (17: 22)			

Table S5 Baseline-adjusted estimates for primary and secondary/exploratory outcomes - supplementary

Outcome	Group	Visit	Estimated mean (95% CI)	Within-group changes (95% CI)	Difference from Control (95% CI)	P-value
		End-of-treatment	17 (14: 21)	-10 (-26: 9)	-1 (-25: 30)	0.913
	Standard	Baseline	19 (17: 22)			
		End-of-treatment	17 (14: 22)	-9 (-26: 12)		
**Polyunsaturated fat intake, g/day***	BCC	Baseline	8 (7: 9)			
		End-of-treatment	7 (6: 9)	-6 (-25: 17)	-9 (-33: 25)	0.561
	Standard	Baseline	8 (7: 9)			
		End-of-treatment	8 (6: 10)	3 (-19: 30)		
**Dietary fibre intake, g/day***	BCC	Baseline	22 (20: 25)			
		End-of-treatment	22 (19: 25)	-4 (-18: 14)	5 (-14: 28)	0.638
	Standard	Baseline	22 (20: 25)			
		End-of-treatment	21 (18: 24)	-8 (-22: 9)		
**Dietary fibre intake, g/10 MJ***	BCC	Baseline	30 (27: 33)			
		End-of-treatment	32 (28: 36)	5 (-8: 20)	9 (-10: 32)	0.356
	Standard	Baseline	30 (27: 33)			
		End-of-treatment	29 (25: 33)	-4 (-17: 11)		
**Added sugar intake, g/day***	BCC	Baseline	51 (43: 61)			
		End-of-treatment	39 (29: 52)	-23 (-41: -1)	-17 (-43: 20)	0.310
	Standard	Baseline	51 (43: 61)			
		End-of-treatment	47 (35: 63)	-7 (-29: 22)		

Table S5 Baseline-adjusted estimates for primary and secondary/exploratory outcomes - supplementary

Outcome	Group	Visit	Estimated mean (95% CI)	Within-group changes (95% CI)	Difference from Control (95% CI)	P-value
**Added sugar intake, g/10 MJ***	BCC	Baseline	68 (60: 77)			
		End-of-treatment	58 (46: 73)	-15 (-33: 6)	-16 (-39: 16)	0.280
	Standard	Baseline	68 (60: 77)			
		End-of-treatment	69 (54: 88)	1 (-20: 28)		
**MET-minutes/week***	BCC	Baseline	2040 (1345: 3092)			
		End-of-treatment	7064 (4395: 11355)	246 (94: 518)	49 (-29: 215)	0.284
		Follow-up	7061 (4191: 11895)	246 (78: 574)	39 (-35: 196)	0.382
	Standard	Baseline	2040 (1345: 3092)			
		End-of-treatment	4734 (2664: 8414)	132 (21: 347)		
		Follow-up	5083 (2962: 8724)	149 (26: 393)		

Estimated means (CI 95%) (left) and baseline corrected difference between groups (CI 95%). *Variable has been log-transformed for analysis and back-transformed for presentation, comparisons are presented as relative differences (%). P-values are between group differences. Abbreviations: BCC, basic carbohydrate counting; E%, percentage of total energy intake; HDL, high-density lipoprotein; MET, metabolic equivalent of task; SD, Standard deviation; Standard, standard dietary treatment.

Table S6 - Delta values for diabetes diet-related quality of life, perceived autonomy support and competencies in diet and diabetes

Outcome	Group	Visit	Median (IQR)	P-value
**DDQOL subscales**				
Dietary satisfaction	BCC	End-of-treatment	12.5 (6.3;18.8)	0.723
	Standard		12.5 (0.0;31.3)	
Dietary benefits	BCC	End-of-treatment	10 (-5; 25)	0.306
	Standard		5 (-5;15)	
Dietary burden	BCC	End-of-treatment	4.7 (-6.3;9.4)	0.282
	Standard		0.0 (-6.3;6.3)	
Social restrictions	BCC	End-of-treatment	0 (0;25)	0.758
	Standard		0 (0;25)	
General perception of diet	BCC	End-of-treatment	0 (0;25)	0.788
	Standard		0 (0;25)	
Mental health	BCC	End-of-treatment	0 (-5;5)	0.368
	Standard		0 (-10;5)	
Vitality	BCC	End-of-treatment	12.5 (6.3;31.3)	0.187
	Standard		12.5 (0.0;18.8)	
**PCDS**	BCC	End-of-treatment	5 (3;9)	0.240
	Standard		3 (1;9)	
**HCCQ**	BCC	End-of-treatment	0.9 (0.0;1.7)	0.575
	Standard		0.6 (0.2;1.2)	

Delta values (median and IQR 25th to 75th percentiles) for changes in summed scores from baseline to end-of-treatment between groups. P-values are between group differences. Abbreviations: BCC, basic carbohydrate counting; DDQOL, diabetes diet-related quality of life; HCCQ, health-care climate questionnaire in relation to dietitian support; PCDS, Perceived competencies in diet and diabetes; Standard, standard dietary treatment.
